# Loss of caveolin-1 alters extracellular matrix protein expression and ductal architecture in murine mammary glands

**DOI:** 10.1371/journal.pone.0172067

**Published:** 2017-02-10

**Authors:** Christopher Thompson, Sahar Rahim, Jeremiah Arnold, Abigail Hielscher

**Affiliations:** Department of Biomedical Sciences, Georgia-Philadelphia College of Osteopathic Medicine, Suwanee, Georgia, United States of America; Oregon Health and Science University, UNITED STATES

## Abstract

The extracellular matrix (ECM) is abnormal in breast tumors and has been reported to contribute to breast tumor progression. One factor, which may drive ongoing matrix synthesis in breast tumors, is the loss of stromal caveolin-1 (cav-1), a scaffolding protein of caveolae, which has been linked to breast tumor aggressiveness. To determine whether loss of cav-1 results in the abnormal expression of matrix proteins, mammary glands from cav- 1-/- and cav- 1 +/+ mice were investigated for differences in expression of several ECM proteins. In addition, the presence of myofibroblasts, changes in the vessel density, and differences in duct number and size were assessed in the mammary glands of both animal models. Using immunohistochemistry, expression of fibronectin, tenascin-C, collagens and αSMA were significantly increased in the mammary glands of cav-1-/- mice. Second harmonic generation revealed more organized collagen fibers in cav-1 -/- glands and supported immunohistochemical analyses of increased collagen abundance in the glands of cav-1 -/- mice. Analysis of the ductal structure demonstrated a significant increase in the number of proliferating ducts in addition to significant increases in the duct circumference and area in cav-1 -/- glands compared to cav- 1 +/+ glands. Differences in microvessel density weren’t apparent between the animal models. In summary, we found that the loss of cav-1 resulted in increased ECM and α-SMA protein expression in murine mammary glands. Furthermore, we found that an abnormal ductal architecture accompanied the loss of cav-1. These data support a role for cav-1 in maintaining mammary gland structure.

## Introduction

Caveolin-1 (cav-1) is the main structural protein of caveolae [1], 50-100nm sized invaginations in the plasma membrane [2], responsible for macromolecular transcytosis [3] and regulation of signal transduction [4, 5]. Cav-1 has been found to be highly expressed in mesenchymal cells including adipocytes, endothelial cells and smooth muscle cells [4] and has been reported to act as a tumor suppressor [6, 7]. In breast cancer, cav-1 is frequently down regulated in fibroblasts of patient breast tumors [8] and has been associated with a more aggressive tumor signature. For example, reduced stromal expression of cav-1 has been associated with a poor prognosis [9, 10], decreased disease-free survival [11], tumor invasiveness [11], lymph node metastases [10], and hormone receptor status [10] in patients with breast cancer. In addition, the loss of stromal cav-1 has also been reported to be a prognostic indicator for early breast tumor recurrence [11]. Mammary glands of cav-1-/- mice have been shown to possess regions of hyperplasia [12], implicating that the loss of stromal cav-1 results in aberrant tissue architecture. In addition, cav-1-/- mice crossed with tumor prone MMTV/PyMT mice were reported to have an accelerated appearance of dysplastic foci in mammary glands [13] and were later shown to have an increased number of lung metastases [14]. Together, these studies indicate that the loss of cav-1 is an important driver of breast tumor progression. Although recent efforts have been aimed at understanding the contribution of cav-1 toward tumor growth, it remains unknown whether the loss of cav-1 is associated with alterations in the stromal architecture of the gland, a phenomenon that has been extensively reported to accompany breast tumor progression.

All cells in the body reside in a supportive structure known as the extracellular matrix (ECM), a non-cellular entity that acts as a scaffold to maintain 3-dimensional (3D) tissue architecture. The ECM actively participates in numerous cellular activities including cell adhesion, survival, proliferation, differentiation and migration in addition to providing mechanical support to overlying cells [15]. Locally resident cells, such as fibroblasts secrete the components that make up the ECM of which the main constituents include collagens and glycoproteins such as fibronectin [16–18]. Production and modification of these ECM proteins are further enriched following fibroblast activation into a proliferative, contractile phenotype. Referred to as myofibroblasts or carcinoma-associated fibroblasts (CAFs), these cells participate in breast tumorigenesis through secretion of cytokines and growth factors and an exaggerated production of ECM proteins [19].

Alterations in ECM composition have been attributed to initiating tumorigenesis [20]. In breast carcinomas, mammographically dense breast tissue is commonly linked to an increased risk of developing breast cancer [21–23]. In particular, it has been shown that total collagen increases with increasing breast density as defined using radiographic measures [24, 25]. Not surprising, several groups have shown a correlation between increased collagen density and breast tumor invasion and metastasis [26–30]. In addition, the glycoprotein fibronectin is also aberrantly expressed in breast tumors and has been associated with tumor initiation [31], invasion and metastasis [32] and therapy resistance [33]. Interestingly, it was found that collagen density and organization in addition to increased fibronectin expression were upregulated in the dermis of cav-1 -/- mice [34]. Furthermore, stabilization of fibronectin fibrils in the ECM was reported to be a result of RNAi-mediated inhibition of cav-1 in fibroblasts [35]. Together, these reports suggest that abnormal matrix protein expression in breast tumors drives disease progression and that caveolin-1 may be an important regulator of matrix protein expression.

Given the roles for the ECM in breast tumor progression and their association with cav-1 expression, we tested the hypothesis that the loss of cav-1 in murine mammary glands was associated with altered expression of stromal ECM proteins and was additionally responsible for disrupting healthy ductal architecture. As such, the goal of this project was to determine whether absence of caveolin-1 resulted in tissue and ductal abnormalities indicative of the early events of breast tumorigenesis. In this paper, we report that the loss of cav-1 resulted in increased expression of the matrix proteins fibronectin, tenascin-C, and collagen I. In addition, expression of alpha-smooth muscle actin (αSMA), a well-characterized marker for myofibroblasts [36], was increased in the myoepithelial compartment of mammary glands from cav-1 deficient animals. Furthermore, ductal architecture was disrupted in the mammary glands of cav-1 deficient animals. This was evidenced from the presence of an increased number of ducts in addition to ducts that exhibited a larger area and circumference and a higher proliferation index. Overall, our results support a role for the loss of cav-1 on altering the stromal composition and ductal architecture of the murine mammary gland. Combined, these results have important implications for a better understanding of how cav-1 participates in the regulation of normal breast tissue architecture and warrants further investigation to determine whether the reduced expression of this protein participates in the early stages of tumorigenesis.

## Methods

### Animal model

Nulliparous female *Mus musculus* C57BL/6J wild-type control (cav-1 +/+) and B6.Cg-Cav1 homologous knockout (cav-1 -/-) animals were obtained from Jackson Laboratories, Bar Harbor, ME. All animals were received between 4–6 weeks of age and were allowed to acclimate to their new colonies for 3–4 weeks before undergoing necropsy for mammary gland isolation. Procedures on animals were performed in accordance with an approved GA-PCOM IACUC.

### Mammary gland harvest and tissue fixation

Prior to mammary gland collections, animals were euthanized using CO_2_ asphyxiation. Animals were subsequently subjected to necropsy wherein the inguinal mammary glands were collected from six animals in each group. Immediately post-harvest, glands were placed in 5–10 mL of 10% neutral buffered formalin (Sigma-Aldrich) for 48–72 hours. Tissues were processed at the Research Pathology, Winship Cancer Institute, Emory University Medical Center, Atlanta, Georgia). Tissues were paraffin block embedded (FFPE) and were sectioned at 5 μm slices onto standard, charged glass slides using an automated microtome.

#### Immunohistochemistry

Slides were de-paraffinized in 100% xylene for 10-minutes. The slides were dehydrated by placing in 100% ethanol for 6-minutes followed by 3-minute incubations each of 95%, 75%, and 50% ethanol. In order to cleave epitopes from sections of tissue, slides were incubated in 10 mM sodium citrate solution (pH adjusted to 6.0) at 95°C for 10-minutes, and then allowed to cool to room temperature for 20-minutes. Once slides were cooled, the endogenous enzyme activity of tissues was blocked using 200 μL dual peroxidase and alkaline phosphatase blocking reagent (Dako EnVision Kit) for 5±1-minutes in a humidified chamber. After a brief wash in deionized water, the slides were incubated in 10% donkey serum (EMD Millipore) for 1 hour. After blocking, the primary antibody was prepared in 1% donkey serum in PBS. Antibodies used include: 1:150 anti-fibronectin (Abcam); 1:50 anti-tenascin-C (Abcam); 1:100 anti-αSMA (Abcam); and 1:25 CD31 (Abcam). Slides were allowed to incubate for 1 hour in a humidified chamber at room temperature or at 4°C overnight. Following prescribed incubations, slides were washed briefly with diH_2_O. Approximately 200 μL of anti-rabbit labeled polymer (Dako EnVision Kit) was applied to the tissues and incubated at room temperature in a humidified chamber for 30 minutes. Immediately after secondary incubation, slides were briefly rinsed with diH_2_O then placed in 1X PBS for 5-minutes. Approximately 200 μL of DAB+ Chromogen solution (Dako) was added to tissues and incubated for 5±1-minutes. Slides were carefully rinsed with diH_2_O and briefly cleared by 100% xylene dip before proceeding to hematoxylin and eosin (H&E) staining or coverslip mounting. For H&E staining, slides were submerged in Harris’s Hematoxylin (Sigma-Aldrich) for 2 to 2.5-minutes. Next, slides were carefully washed in running tap water followed by 2 brief dips in differentiation solution (0.25% hydrochloric acid in 70% ethanol). The slides received a second tap water rinse before a 60-second submersion in a bluing solution (4.5 mg calcium carbonate dissolved in 500 mL of tap water and adjusted pH 9.4). Slides were removed from the bluing solution and placed in a bath of 95% ethanol for 30 seconds. Slides were incubated in Alcoholic Eosin Y (Sigma-Aldrich) for 0.5 to3-minutes. Excess eosin was removed with 1 dip in 95% ethanol and dehydrated in 2 washes of 95% ethanol at 5-minutes each. Sections were mounted with 1–2 drops of aqueous, permanent mounting media (Dako) and visualized using an Olympus BX43 upright microscope at 10, 20 and 40x magnifications. Images were captured using the attached Olympus DP73 color camera and processed using Olympus cellSens Entry software. Six representative stained sections for fibronectin, tenascin-C and αSMA from each animal model at 20x magnification were analyzed for the abundance of protein expression using the IHC toolbox in Image J.

#### Picrosirius red staining

Analysis of the expression of stromal collagen I was achieved by picrosirius red (PSR, Abcam) staining. Sections were de-paraffinized and dehydrated using the steps previously described. Slides were incubated in PSR for 1 hour followed by regressive staining in 2 brief dips in 5% acetic acid solution. Slides were then dehydrated in two washes of 95% ethanol. All slides were mounted using Eukitt’s Mount (Sigma-Aldrich) and cover-slipped. Sections were visualized using an Olympus BX43 upright microscope at 10, 20 and 40x magnification. Images were captured using the attached Olympus DP73 color camera and processed using Olympus cellSens Entry software. Six representative PSR stained sections of each animal model at 20x magnification were analyzed for the abundance of stromal collagens using the IHC toolbox in ImageJ.

#### Intensity measurements

Representative images of each animal model and stain (fibronectin, tenascin-C, αSMA, and PSR) were selected. A total of 6 images at 10x magnification from each stain was analyzed. DAB-associated brown or PSR-associated red staining within each image was automatically isolated using IHC Toolbox plugin for ImageJ. Using ImageJ, the resulting images were converted to a 16-bit image and thresholded to avoid the inclusion of counterstain. The mean grey value was obtained from the resulting images. This value yielded a single number that represented the total stain density divided by the total number of pixels.

#### Second harmonic generation

Collagen organization was analyzed in cav 1 -/- and cav 1 +/+ mammary glands using multiphoton microscopic detection of second harmonic generation (SHG). The acquisition of SHG images was achieved utilizing a Leica TCS SP8 Confocal Laser Scanning Microscope with a 63x oil immersion objective. Photon excitation was induced by a Coherent Chameleon Ultra-II sapphire laser generator while activity was received by 2 PMT and 2 HyD detectors. The multiphoton laser was initially tuned to 800 nm and detectors trained to receive SHG signals between 400–425 nm. Images were captured and processed using Leica LAS X software, with 1x line accumulation, 8x line average, 1x frame average and 2x frame accumulation. Multiple line averages were used in order to increase the resolution of the images and two times frame accumulation was used to increase the visibility of the fibers overall. A total of 5 images per animal model were analyzed.

#### Microvessel density

Differences in vessels numbers were analyzed using a 1:25 dilution of CD31 (Abcam). Slides from cav-1 -/- and cav-1 +/+ glands were prepared for immunohistochemistry and imaged as previously described. Vessels in 20x magnified images were manually enumerated from 3–6 non-overlapping images each in 5–6 slides from cav-1 -/- and cav-1 +/+ glands.

#### Ductal measurements

Using ImageJ software, mammary gland ducts and surrounding stroma were analyzed in 10x images from cav 1+/+ and cav 1-/- mammary gland sections stained with αSMA and H&E. Careful to exclude vascular structures, which were identified by morphological differences including the presence of a tunica media and flattened, elongated cells and nuclei in place of columnar-like cells nuclei bordering the lumen, ducts were counted in representative images (n = 30). To analyze the ductal area and circumference, ducts from the same images used for ductal counts were measured (n = 30). To maintain consistency, only ducts with an intact lumen were analyzed. To examine the ductal circumference, a vector was drawn using Image J (NIH) around the periphery of the myoepithelial compartment. To investigate the ductal area, vectors were drawn using Image J (NIH) to encompass the myoepithelial and luminal compartments. The total area encompassed within the vector drawn around the lumen was subtracted from the vector drawn around the myoepithelial compartment.

### Proliferation analyses

To determine differences in the proliferation of ductal epithelial cells, slides from cav-1 -/- and cav-1 +/+ glands were immunostained with a 1:200 dilution of ki67 (Abcam and Bioss) using methods previously described. Three slides from each animal model with 3–6 images per slide were quantified for differences in ki67 staining using the Cell Counter tool in ImageJ (NIH).

#### Statistical analyses

All measurements were screened for Gaussian distribution before eliminating the outliers (Q = 1%). Analyses of ductal numbers, area, circumference, microvessel density and ECM/myofibroblast markers in cav-1 -/- glands were analyzed for differences against the cav-1 +/+ control glands using student’s t-test in GraphPad Prism v6. All results are reported as the mean ± the SD or SEM. Statistical significance was assigned at p≤0.05 (*), p≤0.01 (**), p≤0.001 (***), and p≤0.0001 (****).

## Results

### Fibronectin is upregulated in cav-1 -/- mammary glands

**Fig 1:**
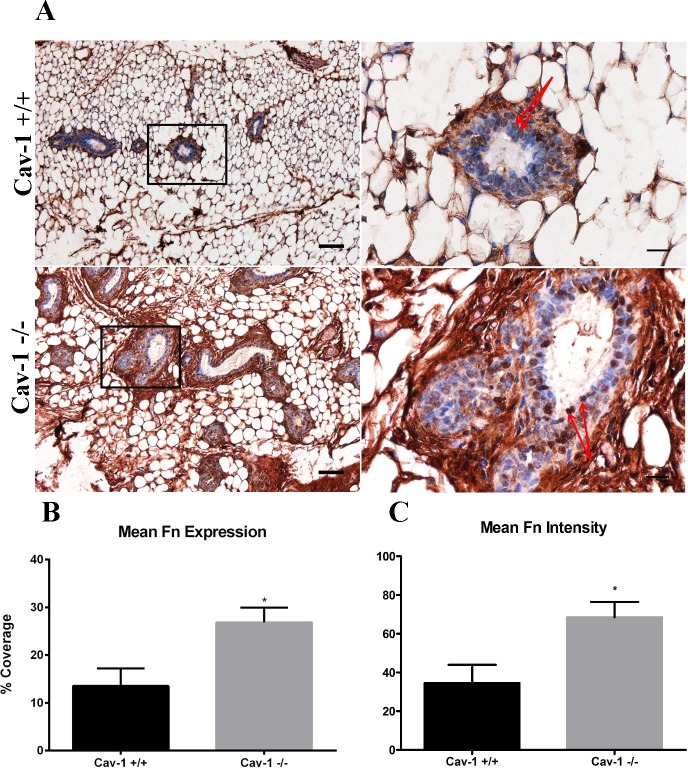
Fibronectin expression is upregulated in cav-1 -/- mammary glands. **(A)** Immunohistochemistry of fibronectin expression in cav-1 +/+ and cav-1 -/- glands at low and high magnification. Results demonstrated an increased abundance fibronectin fibers surrounding the ducts and in the adipose stroma of cav-1 -/- glands. Fibronectin expression was also localized to the epithelial cells (red arrows) of the ducts in both cav-1 +/+ and cav-1 -/- glands. **(B)** The percent expression of fibronectin was significantly higher in cav-1 -/- glands compared to cav-1 +/+ glands. **(C)** The intensity of fibronectin staining was significantly higher in cav-1 -/- glands compared to cav-1 +/+ glands. Scale bars are 50 μM and 20 μM in low and high magnification images, respectively. *p≤0.05. Fn: fibronectin. Results are reported as the mean ± the SEM.

Increased fibronectin expression has been shown to both accompany breast tumorigenesis and support breast tumor progression [32, 37–39]. Furthermore, siRNA of cav-1 in fibroblasts has been shown to result in excess fibronectin matrix deposition [35]. In light of these studies, we sought to first determine whether the loss of cav-1 resulted in increased mammary gland fibronectin expression. Cav-1 -/- glands were generally observed to have prominent increases in the density and abundance of fibronectin (Fig 1A). Fibronectin was located most abundantly in the stroma immediately surrounding the ducts (peri-ductal) and was observed to occupy regions within the adipose stroma as well (Fig 1A). In addition, fibronectin expression was also observed in the epithelial cells lining the ducts (Fig 1A). Cav-1 +/+ glands were observed to contain fibronectin fibers in the peri-ductal and adipose stroma (Fig 1A). Similar to cav-1 -/- glands, some fibronectin expression was also apparent in the epithelial cells lining the ducts (Fig 1A). Overall, the abundance of fibronectin in cav-1 +/+ glands was considerably less than that which was observed for cav-1 -/- glands (Fig 1A). Analyzing the percent coverage of fibronectin, it was found that fibronectin expression was 2-fold more abundant in the mammary glands of cav-1 -/- mice as compared to cav-1 +/+ mice, 26.9% versus 13.6% (p = 0.0206), respectively (Fig 1B). Analyzing differences in staining intensity, it was found that cav-1 -/- glands exhibited a significantly greater degree of staining intensity for fibronectin than cav-1 +/+ glands (Fig 1C), supporting visual and quantitative data that fibronectin staining is more abundant in cav-1 -/- glands. These results demonstrate that the loss of cav-1 promotes the increased expression of peri-ductal and adipose stromal fibronectin in murine mammary glands and further indicates that the overall abundance of fibronectin is greater in cav-1 -/- glands.

### Tenascin-C is upregulated in cav-1 -/- mammary glands

**Fig 2:**
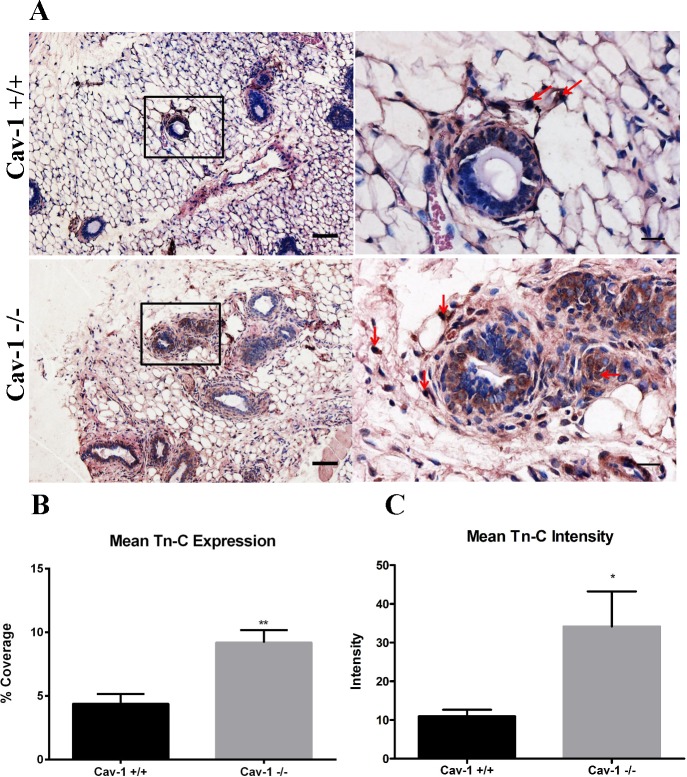
Tenascin-C expression is upregulated in cav-1 -/- mammary glands. **(A)** Immunohistochemistry of tenascin-C expression in cav-1 +/+ and cav-1 -/- glands at low and high magnification. Results demonstrated an increased expression of tenascin-C in the peri-ductal and adipose stroma in cav-1 -/- glands. This is in contrast to cav-1 +/+ glands where tenascin-C expression is expressed in the peri-ductal stroma and to a lesser extent in the adipose stroma. Tenascin-C was also expressed in the stromal cells in cav- 1-/- and cav-1 +/+ glands (red arrows), albeit with a greater proportion of positive cells in cav-1 -/- glands. **(B)** The percent expression of tenascin-C was significantly higher in cav-1 -/- glands compared to cav-1 +/+ glands. **(C)** The intensity of tenascin-C staining was significantly higher in cav-1 -/- glands compared to cav-1 +/+ glands. Scale bars are 50 μM and 20 μM in low and high magnification images, respectively. *p≤0.05, **p≤0.01. Tn-C: tenascin-C. Results are reported as the mean ± the SEM.

Tenascin-C is a stromal protein that has been reported to be more highly expressed in invasive breast carcinomas [40] and has also been shown to support lung metastatic niche formation from breast cancer cells [41]. Although tenascin-C appears to have a prominent role in breast tumor invasion and metastasis, we sought to determine whether the loss of cav-1 promoted increased mammary gland expression of tenascin-C. This is in part due to a recent report that demonstrated that stromal deposition of tenascin-C from fibroblasts co-cultured with breast cancer cells occurred exclusively in the presence of a pre-established cell-derived fibronectin matrix [42]. Results show that cav-1 +/+ glands demonstrated some tenascin-C staining which was primarily confined to the peri-ductal stroma (Fig 2A). Cav-1 -/- glands, on the other hand, exhibited tenascin-C, which was not only expressed in the peri-ductal stroma but also in the adipose stroma (Fig 2A). In addition, tenascin-C also appears to be expressed in some of the stromal cells in cav-1 -/- and cav-1 +/+ glands, with more extensive expression in cav-1 -/- glands (Fig 2A). Comparing the expression of tenascin-C between the two animal models, it was found that tenascin-C was more than 2 fold higher in cav-1 -/- glands in comparison with cav-1 +/+ glands, 9.2% and 4.4%, respectively (Fig 2B). Additionally, cav-1 -/- glands exhibited a significantly greater degree of staining intensity for tenascin-C than cav-1 +/+ glands (Fig 2C). These results indicate that loss of cav-1 upregulates tenascin-C expression primarily in the stroma surrounding and in proximity to the ducts in addition to stromal and epithelial cells and also indicates that the overall abundance of tenascin-C is greater in cav-1 -/- glands.

### Collagen abundance and organization is increased in cav-1 -/- mammary glands

**Fig 3:**
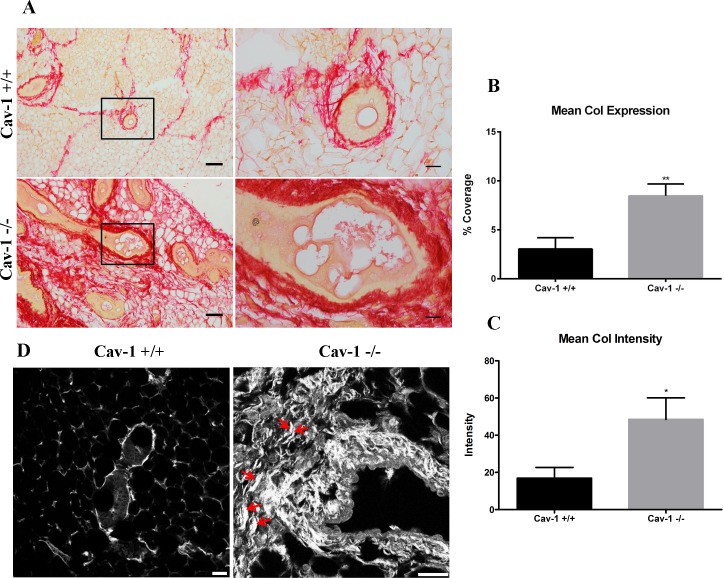
Collagen I expression and organization are upregulated in cav-1 -/- mammary glands. **(A)** PSR staining for collagen I in cav-1 +/+ and cav-1 -/- glands at low and high magnification. Imaging demonstrates extensive expression of collagen I in the adipose stroma and in the peri-ductal region of cav-1 -/- glands. The collagen I fibers appear dense and tightly packed in cav-1 -/- glands. In contrast, overall collagen I expression is considerably lower in cav-1 +/+ glands with fibers being predominantly located to the peri-ductal region. Here, fibers are less packed and distinct fibrils are apparent. **(B)** The percent expression of PSR stained collagen I was significantly higher in cav-1 -/- glands compared to cav-1 +/+ glands. Scale bars are 50 μM and 20 μM in low and high magnification images, respectively. **(C)** The intensity of PSR stained collagen I was significantly higher in cav-1 -/- glands compared to cav-1 +/+ glands. **(D)** Representative SHG images show an abundance of dense, thick collagen fibers in cav-1 -/- glands. Collagen fibers and fiber bundles also appear more organized and aligned (red arrows). Cav-1 +/+ glands express comparably less collagen which presents as thin, unorganized fibers. Scale bars are 25 μM. Col: collagen. *p≤0.05, **p≤0.01. Results are reported as the mean ± the SEM.

Collagen, the most abundant ECM protein in the body, including the breast, has been reported to be increased in the dermis of cav-1 -/- mice [34]. To determine whether collagen is similarly upregulated in the mammary glands of cav-1 -/- mice, immunohistochemistry for collagen was performed using picrosirius red (PSR) which largely detects the birefringence of co-aligned fibers of collagen I. Images from PSR stained slides indicate that glands from cav-1 -/- animals possess extensive stromal expression of collagen in comparison to cav-1 +/+ glands, which exhibit markedly less stromal collagen (Fig 3A). Here, the collagen fibers in cav-1 -/- glands are more densely packed and thicker, especially in the peri-ductal stroma, in comparison to cav-1 +/+ glands, which possess thinner fibers primarily confined to the peri-ductal stroma (Fig 3A). These visual inspections were supported by quantitative analyses wherein loss of cav-1 resulted in an average collagen expression of 8.5% as opposed to 3% in cav-1 +/+ animals (Fig 3B), indicating that collagen is almost 3 fold upregulated in the mammary glands of cav-1 deficient animals. Furthermore, analysis of staining intensity revealed that cav-1 -/- glands exhibited a significantly greater degree of staining intensity for PSR collagen than cav-1 +/+ glands (Fig 3C), supporting quantitative analyses of overall collagen expression and abundance.

To further support findings from PSR data, we sought to examine the abundance and organization of collagen in cav-1 -/- and cav-1 +/+ glands using SHG, a powerful imaging modality which identifies collagen fibers in unstained tissue sections. Using this technique, we were able to which capture both type I and type II aligned collagen fibers [43]. Similar to PSR stained sections, glands from cav-1 -/- animals demonstrated an increase in collagen density that was further accompanied by a distinguishable organization of the collagen fibers (Fig 3D). With regard to organization, cav-1 -/- glands presented with large collagen bundles that were not only aligned (arrows), but also projected into the adipose stroma away from ducts (Fig 3D). In cav-1 +/+ glands, expression of collagen fibers were sparse and were much thinner in comparison to cav-1 -/- glands (Fig 3D). These data demonstrate that loss of cav-1 results in increased expression of organized stromal collagens.

### αSMA is upregulated in cav-1 -/- mammary glands

**Fig 4:**
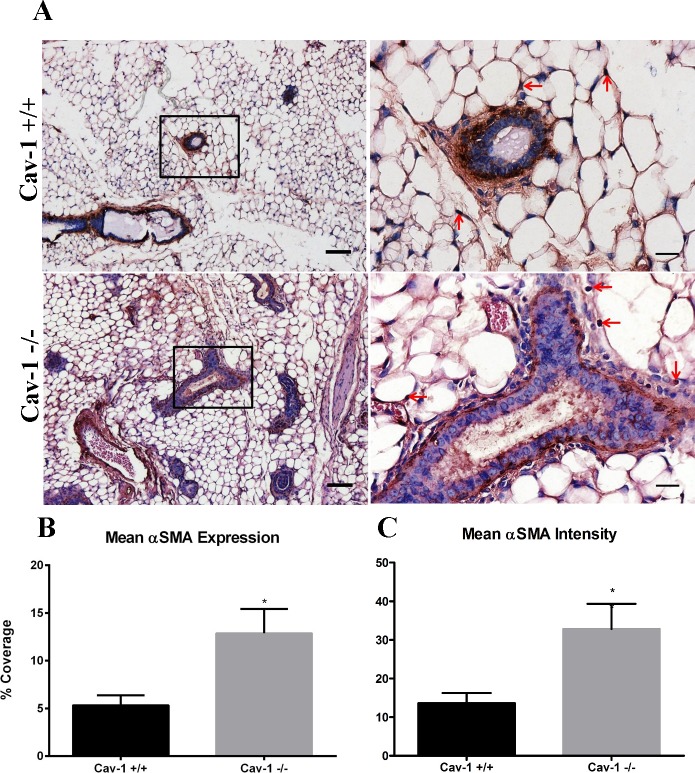
αSMA expression is upregulated in cav-1 -/- mammary glands. **(A)** Immunohistochemistry of αSMA in cav-1 +/+ and cav-1 -/- glands at low and high magnification. Results demonstrated that αSMA expressing cells are predominantly localized to the myoepithelial compartments in cav-1 +/+ and cav-1 -/- glands. αSMA expressing cells are also observed in adipose stromal cells (arrows) in cav-1 +/+ and cav-1 -/- glands **(B)** The percent expression of αSMA was significantly higher in cav-1 -/- glands compared to cav-1 +/+ glands. **(C)** The intensity of αSMA staining was significantly higher in cav-1 -/- glands compared to cav-1 +/+ glands. Scale bars are 50 μM and 20 μM in low and high magnification images, respectively. *p≤0.05. Results are reported as the mean ± the SEM.

αSMA is the well-characterized marker of myofibroblasts or CAFs [36], activated fibroblasts that participate in wound healing and tumorigenesis [44]. It’s been reported that cav-1 deficient fibroblasts have properties similar to breast CAFs [45]. As such, we sought to determine whether αSMA positive stromal cells were upregulated in cav-1 -/- glands. As expected, αSMA was observed to be predominantly localized in the myoepithelial compartment adjacent to the ducts in cav-1 -/- and cav-1 +/+ glands (Fig 4A). Some αSMA staining was observed in stromal cells adjacent to the ducts and was additionally found in cells occupying the adipose stroma in cav-1 -/- and cav-1 +/+glands (Fig 4A). Although there were no discernible differences in αSMA expression between cav-1 -/- and cav-1 +/+ glands, image analysis yielded an average αSMA expression in cav-1 -/- glands at 12.9% compared to 5.3% in cav-1 +/+ glands (Fig 4B). As expected based on visual inspection and quantitative analyses of overall expression, cav-1 -/- glands exhibited a significantly greater degree of staining intensity for αSMA than cav-1 +/+ glands (Fig 4C). Data on stain intensity and overall coverage of tested proteins in cav-1-/- and cav-1 +/+ glands are available in S1 File. These results demonstrate that the loss of cav-1 -/- promotes the increased expression of myoepithelial and stromal cell αSMA, a finding which is presumably due to increased ductal numbers in cav-1 -/- glands.

### Microvessel density is similar in cav-1 -/- and cav-1 +/+ glands

**Fig 5:**
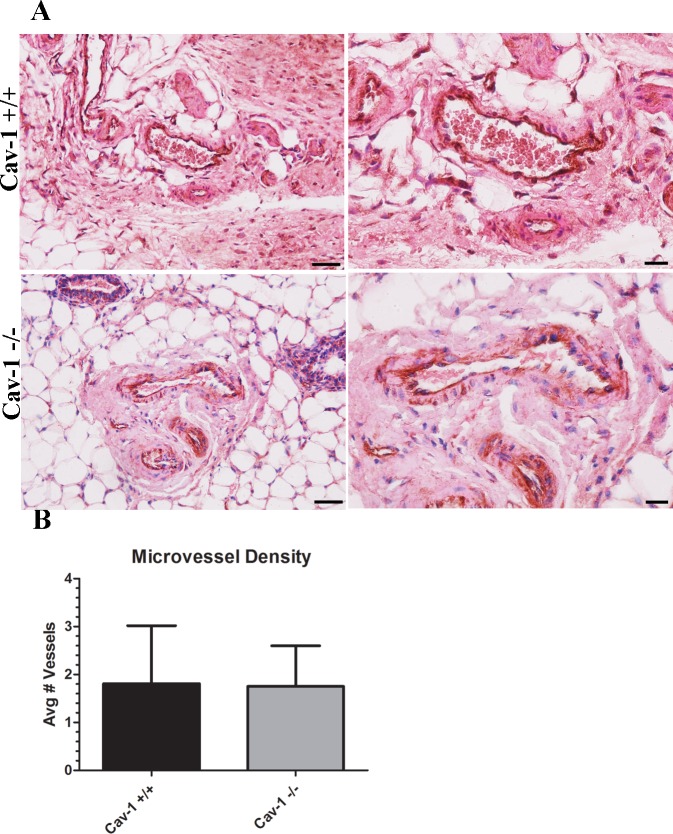
Microvessel density is similar in cav-1 -/- and cav-1 +/+ glands. **(A)** Immunohistochemistry of CD31 expressing endothelial cells in cav-1 +/+ and cav-1 -/- glands at low and high magnification. Results demonstrated that vessels are apparent in both cav-1 +/+ and cav-1 -/- glands. **(B)** Quantification of microvessels show no differences in the abundance of vessels in cav-1 +/+ and cav-1 -/- glands. Scale bars are 50μM and 20μM for low and high magnification images, respectively. Results are reported as the mean ± the SD.

Increased vascularization has been reported to occur in response to ECM protein expression [16, 42, 46]. Given the abundance of ECM proteins observed in cav-1 -/- glands, we sought to determine whether the loss of cav-1 resulted in increased vascularization in the mammary glands. Following immunohistochemistry staining for the endothelial marker CD31 and enumeration of vessels, it was found that there were no differences in the microvessel density between the two animal models (Fig 5A and 5B). In fact, there was an average of 1.8 vessels per slide in both cav-1 -/- and cav-1 +/+ glands (Fig 5B). Data on microvessel density in cav-1-/- and cav-1 +/+ glands are available in S3 File. indicating that neither the loss of cav-1 nor the increased ECM protein expression observed in cav-1 -/- glands promote tissue vascularization.

### Ductal architecture is aberrant in cav-1 -/- mammary glands

**Fig 6:**
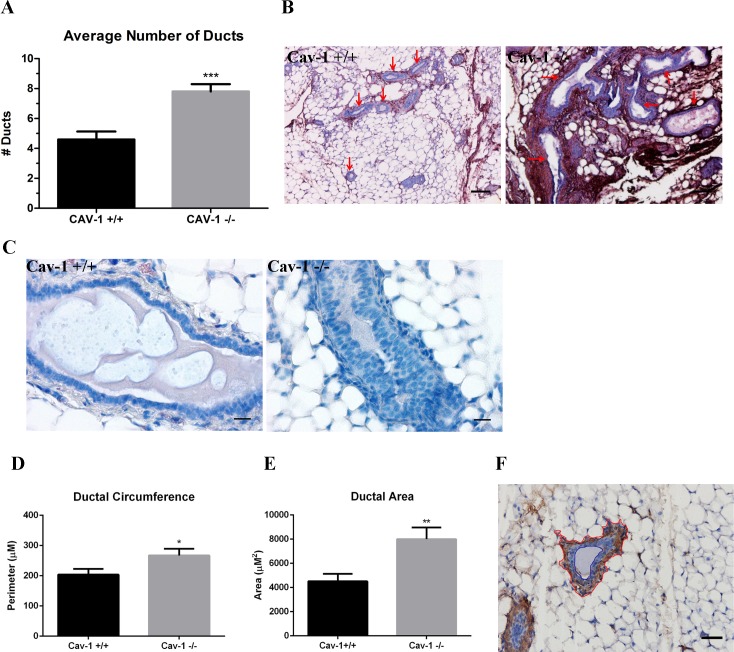
Ductal architecture is disrupted in cav-1 -/- glands. **(A)** The number of ducts were enumerated in αSMA stained sections from cav-1 -/- and cav-1 +/+ glands. Results demonstrated a highly significant average number of ducts per image in cav-1 -/- glands in comparison to cav-1 +/+ glands. **(B)** Images of fibronectin stained sections illustrate the abundance of large, elongated, branched ducts (red arrows) in cav-1 -/- glands. Ducts in cav-1 +/+ glands appear small and relatively round (red arrows). Scale bars are 100μM. **(C)** H&E stained images of glands demonstrate the presence of stacked ductal epithelial cells in cav-1 -/- sections, indicative of ductal hyperplasia. Cav-1 +/+ glands exhibit a continuous monolayer of ductal epithelial cells. Scale bars are 20μM. **(D)** The ductal circumference, measured as the region immediately outside the myoepithelial compartment, was analyzed in αSMA stained sections in cav-1 -/- and cav-1 +/+ glands. The ductal circumference was significantly greater in cav-1 -/- as opposed to cav-1 +/+ glands. **(E)** The area, measured as the region between the myoepithelial and luminal compartment, was analyzed in αSMA stained sections in cav-1 -/- and cav-1 +/+ glands. The ductal area was significantly greater in cav-1 -/- as opposed to cav-1 +/+ glands. **(F)** This image of an αSMA stained cav-1 +/+ duct illustrates the vectors used to assign stromal and lumen outlines used to analyze ductal measurements. The red line shows the stromal border while the blue line shows the lumen border. Scale bar is 50uM. *p≤0.05, **p≤0.01, ***p≤0.001. Results are reported as the mean ± the SEM.

To determine whether the loss of cav-1 resulted in alterations to the ductal architecture, we investigated differences in duct number, circumference and area. Not only were average duct numbers significantly increased in cav-1 -/- versus cav-1 +/+ glands: 7.8 and 4.6, respectively, but ducts in cav-1 -/- glands tended to be highly branched and convoluted in contrast with the round, largely non-branched ducts of cav-1 +/+ glands (Fig 6B). In addition to these morphological differences, ducts in cav 1 +/+ glands mostly presented as single-celled columnar epithelium while the cav-1 -/- glands often had ducts that were large, disorganized, multicellular, and compacted with epithelium lacking the traditional columnar/cuboidal ductal formation, indicative of ductal hyperplasia (Fig 6C). Next, we examined differences in ductal circumference to support observations that cav-1 -/- ducts are larger. We also investigated ductal area to support observations that cav-1 -/- ducts exhibit increased ductal hyperplasia. As expected, significant increases were also observed for the average duct circumference: 267.2μm and 203.8μm (Fig 6D) and area: 8,003μm^2^ and 4,510μm^2^ (Fig 6E) in cav-1 -/- and cav-1 +/+ glands, respectively. To examine the ductal circumference, a red vector was drawn using Image J around the periphery of the myoepithelial compartment (Fig 6F). Similarly, to investigate the ductal area, red and blue vectors were drawn using Image J to encompass the myoepithelial and luminal compartments, respectively (Fig 6F). The total area encompassed within the vector drawn around the lumen was subtracted from the vector drawn around the myoepithelial compartment. The area thus represents the region contained between the myoepithelial and epithelial cells lining the lumen. Data on ductal metrics in cav-1-/- and cav-1 +/+ glands are available in S1 File. These results indicate that cav-1 -/- ducts are not only more numerous and branched having distinct patches of hyperplasia, but are larger than wild type cav-1 +/+ ducts.

**Fig 7:**
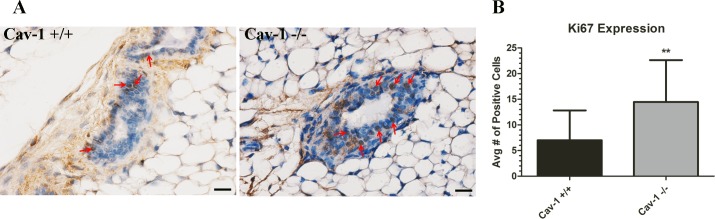
Proliferation of ductal epithelial cells is increased in cav-1 -/- glands. **(A)** Immunohistochemistry of ki67 cells in cav-1 +/+ and cav-1 -/- glands at low and high magnification. Results from representative images demonstrated that ki67 expressing ductal epithelial cells (arrows) are markedly increased in cav-1 -/- glands in comparison to cav-1 +/+ glands. **(B)** The average number of ki67 positive cells was significantly higher in cav-1 -/- glands in comparison to cav-1 +/+ glands. Scale bars are 20uM. **p≤0.01. Results are reported as the mean ± the SD.

In an effort to determine whether the aberrant ductal architecture was a result of increased ductal epithelial cell proliferation, cav-1 -/- and cav-1 +/+ glands were stained for the proliferation marker ki67. Immunohistochemistry findings demonstrated an increased proportion of positively stained ki67 ductal cells in cav-1 -/- as opposed to cav-1 +/+ glands (Fig 7A). To quantify the number of proliferating ductal epithelial cells in cav-1 -/- and cav-1 +/+ glands, ki67 expressing cells were enumerated using the Cell Counter tool in ImageJ. Results demonstrate a statistically significant higher proportion of ki67 positive ductal epithelial cells in cav-1 -/- glands in comparison to cav-1 +/+ glands (Fig 7B). Data on the numbers of ki67 positive cells in cav-1-/- and cav-1 +/+ glands are available in S3 File. These data suggest that the loss of cav-1 supports an environment conducive to ductal proliferation.

## Discussion

Breast cancer is a complex disease of heterogeneous origins. One mechanism that facilitates breast tumorigenesis is decreased expression of stromal cav-1. Previous studies have shown that downregulation of cav-1 in breast cancer has led to cancer invasion and metastasis [10, 11, 47], poor clinical outcome [48], altered therapy responses [49], and decreased progression-free survival time [10, 11]. In addition to cav-1, altered ECM protein expression has been shown to facilitate breast tumorigenesis. For example, increased fibronectin, collagen and tenascin-C accompany breast tumorigenesis and participate in tumor progression, invasion and metastasis [28, 29, 32, 37–41, 50–52]. Since the development and progression of breast cancer are dependent on changes in the ECM, we sought to investigate how ECM protein expression and organization are altered in response to the loss of cav-1. Here, we provide evidence that the loss of cav-1 resulted in increased expression of collagens, fibronectin, and tenascin-C in addition to the myoepithelial and myofibroblast marker αSMA in murine mammary glands. In addition, we also demonstrate that the loss of cav-1 contributes to an altered ductal architecture, characterized by an increased number of ducts, which were more proliferative and hyperplastic and exhibited a larger circumference and area in comparison to wild-type controls. Combined, these results support a role for the loss of cav-1 in altering mammary gland ECM composition and ductal morphology.

Since aberrant fibronectin expression has been shown to support breast tumorigenesis, we sought to first determine whether the loss of cav-1 increased the stromal expression of this matrix protein. We found that fibronectin was more than 2-fold enriched in cav-1 -/- glands as opposed to cav-1 +/+ glands and that the intensity of staining was also significantly increased in cav-1 -/- glands. In cav-1 -/- glands, the fibronectin fibers appeared dense and widespread throughout the adipose stromal tissue and in the peri-ductal stroma. Cav-1 +/+ glands also exhibited fibronectin fibers in the peri-ductal stroma and adipose stromal tissue; however, the abundance of these fibers was considerably less than that observed for cav-1 -/- glands. These data support previous work showing that RNAi of cav-1 in murine fibroblasts promoted increased matrix fibronectin deposition and decreased fibronectin degradation, a result that was dependent on the absence of cav-1 mediated endocytosis of fibronectin [35]. Given these findings in addition to other work demonstrating that fibronectin co-localizes with cav-1 in lipid rafts [53], it’s likely that the increased abundance of fibronectin observed in cav-1 -/- glands is a result of deficiencies in cav-1 mediated endocytosis of fibronectin. Although a likely possibility, it will be necessary to validate that this mechanism not only takes place in the mammary gland, but that it occurs *in-vivo* as well. Of interest is the examination of whether the degree of stromal cav-1 loss coincides with increased fibronectin expression in breast tumors. Presumably, the more severe decrease in stromal cav-1 would result in the greatest overall increase in tumor fibronectin expression. Given the prominent roles for the loss of cav-1 and the increase in fibronectin during breast tumorigenesis, it will be important for future work to consider the mechanism whereby cav-1 contributes to fibronectin expression and how the two proteins collaborate during tumorigenesis.

Similar to fibronectin, stromal collagens have been reported to participate in breast tumorigenesis [28, 29, 51]. To investigate collagen expression patterns, we used PSR, which gives a red birefringence signal for more cross-linked collagen (e.g. more mature) and a yellow/green birefringence for less cross-linked collagen (e.g. less mature). Examining collagen expression in cav-1 -/- and cav-1 +/+ glands, we observed that both cav-1 -/- and cav-1 +/+ sections exhibited a red birefringence indicative of the presence of mature, cross-linked collagen I. Loss of cav-1, however, correlated with an almost 3-fold increase in overall collagen I expression and further resulted in a significant increase in staining intensity in comparison to cav-1 +/+ glands. Interestingly, the collagen I fibers were not only more abundant in the peri-ductal and adipose stroma, but were more compact in cav-1 -/- glands as opposed to cav-1 +/+ glands in which collagen I was primarily localized as thin distinguishable fibers in the peri-ductal stroma. This finding is in support of work by Castello-Cros [34], who similarly observed tightly packed collagen fibers in the dermis of cav-1 -/- mice. To determine whether collagen fibers were more aligned in cav-1 -/- glands, a morphological feature associated with breast tumorigenesis, we used SHG, a powerful imaging modality which enables visualization of type I and II collagen fibers in unstained tissue sections [43]. Using SHG, we demonstrated that collagen fibers were sparse and primarily localized to small regions immediately surround the ducts in cav-1 +/+ glands. In cav-1 -/- glands, collagen fibers were not only more abundant but also appeared linearized, a finding which has previously been found to increase matrix stiffness and sequestration of tumor-promoting cytokines in addition to supporting breast tumor progression [27, 28, 54]. It’s likely that collagen fiber organization accompanies increased collagen density as Fraley et al [55] used 3D self-assembling collagen gels to demonstrate that increased collagen density not only supported collagen fiber alignment, but also supported cell motility. As such, it’s possible that both the increased density and organization of collagens in cav-1 -/- glands is setting up a supportive environment for aberrant cell behaviors potentially leading to tumorigenesis. Indeed, it’s well known that women with increased collagen density have a higher risk of developing breast cancer [22, 23, 56]. It will be important for future studies to determine whether the increased collagen density and organization resulting from the loss of cav-1 contributes to abnormal mammary cell behaviors.

In addition to fibronectin and collagen, we also investigated the expression of tenascin-C, a matrix protein shown to support breast tumor invasion [40] and lung metastatic niche formation [41]. As expected, tenascin-C was upregulated in cav-1 -/- glands as analyzed from overall abundance and intensity of staining with expression localized to the peri-ductal and adipose stroma. In cav-1 +/+ glands, tenascin-C staining was primarily confined to the peri-ductal stroma. In prostate cancer, it has been shown that RNAi of cav-1 in benign prostatic myofibroblasts promotes tenascin-C gene expression [57], supporting a role for reduced or absent stromal cav-1 in increased tenascin-C. Although it’s unknown as to mechanism responsible for upregulated tenascin-C in response to cav-1 deficiency, it’s been shown that tenascin-C is deposited in cell-derived ECM in response to a pre-established fibronectin matrix [42]. It’s possible that the presence of excess stromal fibronectin in cav-1 -/- glands acts as a supportive scaffold for the deposition of tenascin-C. It’s also possible that like fibronectin, the loss of cav-1 prevents endocytosis of tenascin-C. Given that the abundance of tenascin-C was markedly less than that observed for fibronectin, one or more mechanisms may be responsible for its increased expression in cav-1 -/- glands. Further work will be necessary to delineate a mechanism(s) responsible for the upregulated tenascin-C expression.

αSMA is the well-characterized marker of myofibroblasts or CAFs [36], activated fibroblasts which not only deposit copious ECM proteins and remodel these but also participate in tumorigenesis [44, 58]. Furthermore, it has been reported that cav-1 deficient fibroblasts have properties similar to breast CAFs [45]. As such, we expected to observe an increased abundance of αSMA expressing cells in the adipose stroma of cav-1 -/- glands, a finding which wasn’t supported. Despite a more than 2-fold increase in abundance and a significant increase in staining intensity for αSMA in cav-1 -/- glands, there were no detectable differences in staining patterns between the two tissues. For instance, we found that αSMA was primarily localized to the myoepithelial compartment, an expected observation given the presence of smooth muscle cells surrounding the ducts, in both animal models. In addition, αSMA was expressed in adipose stromal cells of both animal models. A possible explanation for our discordant observations is the presence of additional ducts that were observed in cav-1 -/- glands. Given the increased number of ducts and thus myoepithelial cells surrounding these ducts, it would be expected that αSMA would be concordantly increased in cav-1 -/- glands.

ECM proteins, especially fibronectin, collagen I, and tenascin-C, have been reported to promote angiogenesis [16, 42, 46, 59]. Given the abundance of these ECM proteins observed in cav-1 -/- glands, we expected that the microvessel density would be correspondingly enriched. Contrary to our expectations, we didn’t find an increased microvessel density in the glands of cav-1 -/- glands but rather observed that the average number of vessels in both animal models was the same. Initially, this observation was surprising given that pro-angiogenic factors such as VEGF and PDGF were reported to be upregulated in the conditioned media from cav-1 -/- murine mammary fibroblasts [45]. Others have found that cav-1 loss resulted in reduced angiogenesis. Specifically, it was shown that vessel density and tumor growth were impaired following subcutaneous injection of melanoma or prostate cancer cells [60, 61]. Based on these xenograft studies, it’s apparent that cav-1 expression is needed in order to support tumor angiogenesis. Although cav-1 -/- fibroblasts were reported to produce greater quantities of pro-angiogenic cytokines, it’s possible that morphological and subsequent functional deficits in cav-1 -/- endothelial cells prevent these cells from responding to fibroblast-derived pro-angiogenic factors. While we didn’t investigate morphological or functional differences in cav-1 -/- and cav-1 +/+ endothelial cells, our results nonetheless support prior work that the loss of cav-1 doesn’t support enhanced tissue angiogenesis.

Since aberrant patterns of ECM protein expression have been shown to alter mammary epithelial behaviors [31], we sought to determine whether changes in ductal morphology were evident in cav-1 -/- glands. We found that cav-1 -/- glands exhibited significant increases in duct numbers as well as greater ductal circumferences and areas than cav-1 +/+ glands. The ducts of cav-1 -/- animals were further found to not only possess an increased proportion of proliferating ki67 positive cells but were also noted to exhibit regions of ductal hyperplasia, characterized by stacked epithelial cells protruding into the lumen. Previously, it has been shown that cav-1 -/- KO mice present with mammary gland hyperplasia and actively proliferating ductal epithelial cells as assessed using bromodeoxyuridine, confirming our findings [62]. Although the increased proportion of proliferating ductal epithelial cells may explain the higher ductal numbers, area and hyperplasia, this does not fully support a role for ductal proliferation in circumference. The increased circumference of the ducts in cav-1 -/- glands suggests that ducts are more elongated. In the pubertal mammary glands of mice, the orientation of collagen fibers determined the directionality of branching ducts, suggesting that collagen organization imparts important cues to the developing ducts of the mammary glands [63]. It’s possible that the increased density and organization of collagen in cav-1 -/- animals may similarly provide directional cues for proliferating ductal epithelial cells, supporting the abnormal outgrowth of these ducts. In addition to collagen, it’s possible that fibronectin is also participating in aberrant ductal cell behavior. For example, Williams et al [31] demonstrated that the addition of fibronectin to cultures of mammary epithelial cells grown atop a basement membrane extract stimulated cell proliferation and reversed the acinar morphology, suggesting that fibronectin disrupts normal ductal architecture. Additional work will be necessary to elucidate the contributions of collagen and/or fibronectin to aberrant ductal organization.

In conclusion, we have shown that loss of cav-1 increases the expression of fibronectin, collagen I, tenascin-C and αSMA in murine mammary glands. Furthermore, loss of cav-1 contributed to a significant increase in the number of ducts exhibiting hyperplasia and a higher proliferation index in addition to ducts having a larger circumference and area. It is well known that the risk for development of breast cancer increases with increasing mammographic density [22, 23, 56] and that increases in stromal proteins including fibronectin and collagen have been implicated in greater tissue stiffness in tumors [64]. Since cav-1 loss results in increased expression of these proteins in murine mammary glands, it’s likely that this aberrant ECM may contribute to a situation which favors the early events leading to tumor initiation. In addition, this abnormal ECM could potentially instigate changes in the complex signaling cascades within the tissue microenvironment that regulate cell proliferation, cellular trans-differentiation and matrix deposition and turnover, further favoring an environment supportive of tumor initiation. As a result, cav-1 expression could potentially be a useful tumor marker to aid clinicians in better diagnoses, more accurate prognoses, and may be a powerful tool in determining the best course of therapy for patients. In summary, our results shed light on a mechanism regulating both the expression of ECM proteins and ductal organization in the murine mammary gland and have important implications for our understanding of the events which may take place during the early stages of breast tumorigenesis.

## Supporting information

S1 File Intensity, Stain Coverage, Duct Metrics. Data are provided regarding the values obtained for immunohistochemistry stain intensity and coverage in addition to data on duct metrics in cav-1 -/- and cav-1+/+ glands.
(XLSX)

S2 File ki67 Analyses. Data are provided regarding the values obtained for analyses of proliferation in cav-1 -/- and cav-1+/+ glands.
(XLSX)

S3 File Microvessel Density. Data are provided regarding the values obtained for analyses of microvessel density in cav-1 -/- and cav-1 +/+ glands.
(XLSX)
